# Comparison of laparoscopic and open approach in the treatment of heterotopic pregnancy following embryo transfer

**DOI:** 10.3389/fsurg.2022.1006194

**Published:** 2022-11-01

**Authors:** Shengfu Chen, Yingying Zhu, Meiqing Xie

**Affiliations:** ^1^ Department of Obstetrics and Gynecology, Sun Yat-sen Memorial Hospital, Sun Yat-sen University, Guangzhou, China; ^2^ Clinical Research Design Division, Clinical Research Center, Sun Yat-sen Memorial Hospital, Sun Yat-sen University, Guangzhou, China

**Keywords:** heterotopic pregnancy, laparoscopy, open surgery, perioperative outcome, pregnancy outcome

## Abstract

**Purpose:**

Heterotopic pregnancy (HP) is a rare disease with the coexistence of an intrauterine and ectopic embryos. There is no consensus on the optimal treatment of HP at present. This research aimed to compare the perioperative and pregnancy outcomes of laparoscopic (LA) and open approach (OA) in patients with HP after embryo transfer.

**Methods:**

Women with HP receiving surgical treatment (LA or OA) were retrospectively recruited in this study between October 2006 and December 2020. The demographic, perioperative and obstetric data were collected and compared between LA and OA group.

**Results:**

Totally, 86 patients were included in this study. Among these patients, 62 underwent LA and 24 underwent OA. There was an increase in the adoption of LA between the 2006–2012 period and the 2013–2020 period [25% (6/24) vs. 90% (56/62), *p* < 0.001]. Compared with OA, patients treated by LA had much less blood loss [20 (10–50) vs. 30 (20–50) ml, *p* = 0.036] and fewer days of hospital stay [5.0 (4.0–7.3) vs. 9.5 (7–15.3) days, *p* < 0.001], but a relatively higher cost (15,580 ± 3196¥ vs. 11,717 ± 3820¥, *p* < 0.001). During the laparoscopic procedure, no one needed to be converted to open surgery. However, the rates of first trimester miscarriage, preterm, cesarean section, birth weight, 1- and 5-min Apgar were similar between LA and OA group (all *p* > 0.05).

**Conclusions:**

Compared with open approach, laparoscopy was shown to provide a comparable pregnancy outcomes and a better performance on perioperative outcomes in the treatment of HP patients with embryo transfer.

## Introduction

Heterotopic pregnancy (HP) is defined as the coexistence of an intrauterine and ectopic embryos (1). HP rarely occurs with an incidence of 1/7,000–1/30,000 of natural conception (2, 3). However, the risk of HP is substantially increased in women with assisted reproductive techniques (ART). The prevalence of HP in women with ART ranged from 1/100 to 1/500 (4, 5). Compared with those with ectopic pregnancy (EP), women with HP are at a greater risk of hypovolemic shock (1). The timely and effective treatment for HP is necessary to ensure the safety of the mother and intrauterine fetus (6, 7).

Currently, there are no clinical guidelines or expert consensus to provide the standard management recommendation for HP due to its rarity. Surgery has been the most common treatment of choice in patients with HP, especially for those with unstable clinical sign or EP ruptures (8–11). Open approach (OA) is the traditional surgical method for HP. With the advance of technology, laparoscopic approach (LA) has attracted more attention from clinicians because of its better visualization and exposure, shorter hospital stays, and less suffering (12, 13). However, the question about the preferred surgical approach for HP patients remains unsolved. Only few case-series and case-report studies have demonstrated that LA could be safely performed in HP patients (10, 11, 14), even for heterotopic interstitial or cornual pregnancies (15–18). In contrast, several studies have found that LA would increase the risk of fetal loss in HP patients (7, 19). The evidence in previous studies were limited by small sample size and lack of comparison between LA and OA in perioperative and pregnancy outcomes of HP patients.

Therefore, the aim of this study was to compare the safety and efficacy of LA and OA in patients with HP after embryo transfer.

## Materials and methods

### Patients

**Figure 1:**
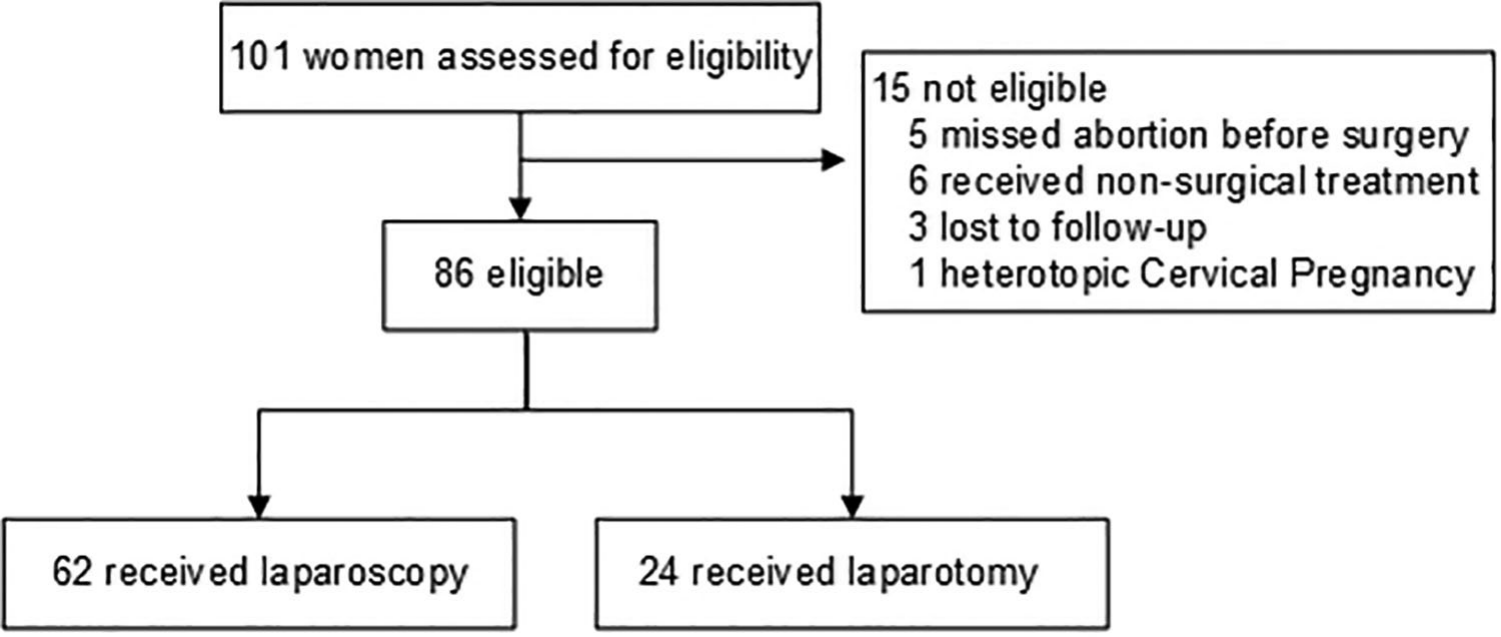
Flow chart of the selection of study participants.

This retrospective cohort study was conducted at Sun Yat-Sen Memorial Hospital between October 2006 to December 2020. Women with HP following embryo transfer and receiving surgical treatment (LA or OA) were enrolled in this study. The diagnosis of HP was confirmed based on both transvaginal ultrasonography (TVS) and histopathological examination. Patients will be excluded if the intrauterine fetal was failed before surgery, or diagnosed with non-tubal HP or lost to follow-up without the perioperative or obstetric data. There were 101 women diagnosed with HP following embryo transfer, among which, 15 patients were excluded. Finally, 86 patients were included in this study. The details of selection is shown in Figure 1.

The study has obtained ethical approval from local ethical committee (Approval number: SYSEC-KY-KS-2020–044). Informed consent was obtained from each patient after explaining the benefits, harms, and possible morbidities of surgery in detail.

### Surgical procedures

The surgical indications for HP in our department were hemodynamic instability, signs of intraperitoneal bleeding, suspected rupture of EP (sudden or persistent abdominal pain) or detectable fetal heartbeat of EP, or an ectopic mass diameter larger than 3 cm or progressively increasing.

Surgeries were performed by surgeons with more than eight years of surgical experience under general anesthesia or spinal anesthesia. Vital signs of patients such as blood pressure, electrocardiogram, blood oxygen saturation and end-tidal CO_2_ pressure were constantly monitored. Laparoscopic and open resections of ectopic mass were performed following the same principles and procedures. During laparoscopy, the abdomen was inflated with CO_2_ while maintaining a pneumoperitoneum pressure of 10–13 mmHg. Salpingectomy was conducted for tubal non-interstitial pregnancy, and cornuostomy for interstitial pregnancy with harmonic scissors. In order to control bleeding of interstitial surgery, a Vicryl loop was sometimes placed laterally to the pregnancy as reported by Soriano et al. (20). The cornual defect was repaired with simple interrupted stitches using a 2–0 synthetic, absorbable, Vicryl sutures (Ethicon, lnc) to reduce the risk of uterine rupture during the ongoing pregnancy.

### Data collection

The data of demographic and clinical characteristics at baseline, perioperative and obstetrical outcomes of all patients were collected *via* medical records or telephone follow-up. The baseline characteristics included age (years), gestational age (days), indication for ART (with vs. without tubal factor), clinical manifestations (vaginal bleeding, abdominal pain), heartbeat of Intrauterine pregnancy (IUP) (with vs. without), site of EP (interstitial vs. non-interstitial), abortion history (yes vs. no), history of pelvic adhesions (yes vs. no) and surgical history (yes vs. no).

The operative time, blood loss, preoperative and postoperative hemoglobin levels were recognized as perioperative outcomes. Besides, the record of blood transfusion, the conversion from laparoscopy to open surgery, and hospital stays were also collected from medical records. Early miscarriage was defined as first trimester fetal loss before gestational age of 14 weeks. Among patients with live birth, delivery mode (cesarean section vs. vaginal deliver), postpartum hemorrhage (yes vs. no), the birth weight, and 1 min and 5 min Apgar for infants were also considered as obstetrical outcomes.

### Statistical analysis

Continuous variables were presented as mean with standard deviation, or median with quartile according to the results of the Kolmogorov–Smirnov test and categorical data as numbers (percentages). Chi-square test or Fisher exact test for categorical variables (treatment period, abortion history, pelvic adhesions, surgical history, history of EP, indication for ART, clinical manifestations, site of EP, yolk sac of EP and heartbeat of EP) and the student's *t*-test (age) or Mann–Whitney *U* test (gestational age) for continuous variables were used to evaluate the difference between laparoscopy and open surgery group, respectively. A *p* value of < 0.05 (two-sided) was considered statistically significant. SPSS 22.0 software (IBM Corporation, Amonk, NY, USA) was used to analyze all data.

## Results

### Patients' characteristics

Among 86 patients with HP after embryo transfer, 62 underwent LA and 24 with OA. The demographic and clinical characteristics of these two groups is shown in Table 1. 68 patients had clinical manifestation (56 only with vaginal bleeding, 7 only with abdominal pain and 5 with both), while the remaining 18 were asymptomatic at admission. Among all HP patients, only two had intrauterine twin gestation. One patient had a live birth after selective fetal reduction, while another one gave live twin birth. There were no statistically significant differences between LA and OA (all *p* > 0.05), except for age and treatment period. Patients with OA were younger than those with LA (29.2 ± 3.4 vs. 31.3 ± 3.8, *p* = 0.014). The adoption of laparoscopic treatment of HP was significantly increased from the period of 2006–2012 (6/24, 25%) to the period of 2013–2020 (56/62, 90%) (*p* < 0.001).

**Table 1:** Baseline characteristics of patients in two groups.

Variables	Laparoscopy	Laparotomy	*P* value
	(*n* = 62)	(*n* = 24)	
Age (year)	31.3 ± 3.8	29.2 ± 3.4	0.014^a^
Treatment period			<0.001
2006–2012	6 (10%)	18 (75%)	
2013–2020	56 (90%)	6 (25%)	
Gestational age (days)	47 (42.8–49.0)	45.5 (40.3–51.0)	0.802^b^
Abortion history			0.246
No	37 (60%)	11 (46%)	
Yes	25 (40%)	13 (54%)	
Pelvic adhesions			0.209
No	9 (15%)	7 (29%)	
Yes	53 (86%)	17 (71%)	
Surgical history			0.687
No	28 (45%)	12 (50%)	
Yes	34 (55%)	12 (50%)	
History of EP			0.708
No	44 (71%)	18 (75%)	
Yes	18 (29%)	6 (25%)	
Indication for ART			0.752
Tubal factor	46 (74%)	17 (71%)	
Non-tubal factor	16 (26%)	7 (29%)	
Clinical manifestations			0.545
Yes	48 (77%)	20 (83%)	
No	14 (23%)	4 (17%)	
Heartbeat of IUP			0.993
With	55 (89%)	22 (92%)	
Without	7 (11%)	2 (8%)	
Site of EP			0.752
Interstitial	16 (26%)	7 (29%)	
Non-interstitial	46 (74%)	17 (71%)	
Yolk sac of EP			0.642
Present	37 (60%)	13 (54%)	
Absent	25 (40%)	11 (46%)	
Heartbeat of EP			0.221
With	27 (44%)	7 (40%)	
Without	35 (56%)	17 (52%)	

IVF-ET, In vitro fertilization and embryo transfer; IUI, intrauterine insemination; ART, assisted reproductive technology; IUP, intrauterine pregnancy; EP, ectopic pregnancy.

^a^ student’s *t*-test.

^b^ Mann–Whitney *U* test; other variables were tested by χ^2^ test.

### Perioperative outcomes

Table 2 presents a comparison of perioperative outcomes between the two groups. Compared with those in the OA group, patients in the LA group had less blood loss [20 (10–50) vs. 30 (20–50) ml, *p* = 0.036] and fewer days of hospital stay [5.0 (4.0–7.3) vs. 9.5 (7.0–15.3) days, *p* < 0.001], but a relatively higher cost (15,580 ± 3196¥ vs. 11,717 ± 3820¥, *p* < 0.001). The surgical indications, hemoglobin levels before and after surgery, as well as changes in hemoglobin level, the operative time, and the percentage of patients receiving blood transfusion were not significantly different between LA and OA group (all *p* > 0.05). No conversion from LA to OA was performed.

**Table 2:** Perioperative outcomes in laparoscopy and open surgery groups.

Category	Laparoscopy (*n* = 62)	Open surgery (*n* = 24)	*P* Value
Surgical indications			0.669^c^
Hemodaynamic instability	4	2	
Suspected rupture/bleeding	11	5	
Hearbeat of EP	27	7	
Mass diameter ≥ 3 cm	20	10	
Operative time, min	70.4 ± 23.5	78.0 ± 31.8	0.231^a^
Intraoperative blood loss (ml)	20 (10–50)	30 (20–50)	0.036^b^
Preoperative HGB (g/L)	118.9 ± 16.2	113.9 ± 17.3	0.221^a^
Postoperative HGB (g/L)	110.8 ± 12.9	110.0 ± 17.0	0.596^a^
Change in HGB (g/L)	−7.0 (−16∼−2)	−8.5 (−13.5∼1)	0.432^b^
Blood transfusion	4 (7%)	4 (16%)	0.367^c^
Hospital stays (day)	5.0 (4.0–7.3)	9.5 (7.0–15.3)	<0.001^b^
Overall expenditures (¥)	15580 ± 3196	11717 ± 3820	<0.001^a^

HGB: hemoglobin; data are presented as median and range.

^a^ student’s *t*-test.

^b^ Mann–Whitney *U* test.

^c^ χ^2^ test.

### Obstetrical outcomes

Pregnancy outcomes are listed in Table 3. After surgery, there were no significant differences in the rates of miscarriage [6% (4/62) vs. 4% (1/24), *p* > 0.99]. Among patients with live birth, the birth weight (3.20 ± 0.55 vs. 2.93 ± 0.47 kg, *p* = 0.06), 1 min Apgar (9 (9–10) vs. 9 (8–9), *p* = 0.14) and 5 min Apgar (10 (10–10) vs. 10 (10–10), *p* = 0.31) of the infants were also similar in the laparoscopic and open surgery group. Also, there were no differences in the delivery mode (*p* = 0.92) between the two groups. No congenital abnormalities were observed.

**Table 3:** Obstetric outcomes in laparoscopy and open surgery groups.

Category	Laparoscopy (*n* = 62)	Open surgery (*n* = 24)	*P* value
Pregnancy outcome			>0.99^a^
Miscarriage	4 (6%)	1 (4%)	
Live birth	58 (94%)	23 (96%)	
Delivery mode			0.92^a^
Cesarean section	36 (62%)	14 (61%)	
Vaginal delivery	22 (38%)	9 (39%)	
Postpartum hemorrhage	2 (3%)	0	>0.99^b^
Birth weight (kg)	3.20 ± 0.55	2.93 ± 0.47	0.06^c^
1 min Apgar	9 (9–10)	9 (8–9)	0.14^d^
5 min Apgar	10 (9–10)	10 (10-10)	0.31^d^

^a^ χ^2^ test.

^b^ Fisher’s exact test.

^c^ student’s *t*-test;

^d^ Mann–Whitney *U* test.

## Discussion

In this retrospective cohort study, we found that the LA had less blood loss, shorter hospital stays and comparable obstetrical outcomes than OA in women with HP after after embryo transfer.

The standard management for HP remains unknown due to the rarity of this disease. Surgical treatment is the most frequently choice for HP (8–11). With the development of equipment and techonology, clinicians have put more and more attention to laparoscopy, rather than open surgery (21–23). In the present study, LA was performed for patients with HP in only 25% (6/24) of cases during the 2006–2012 period. This proportion significantly increased to 90% (56/62) during 2013–2020 period, which was consistent with the result reported in a review (24).

However, there are inconsistent conclusions on the efficacy and safety of LA no matter for treatment of HP or pregnancy with surgical diseases, such as appendicitis and cholecystitis. Several studies have found for patients with appendicitis, an increased rate of fetal loss in the laparoscopic appendectomy compared with the open appendectomy (25–27). Nevertheless, some studies reported that laparoscopic cholecystectomy was associated with decreased risks for fetal, maternal, and surgical complications in patients with symptomatic cholelithiasis (28, 29). Besides, in gynecologic field, several case report or case-series studies have indicated that the feasibility and safety of laparoscopic approach in HP patients. Jiang et al. reported the obstetric outcomes of 7 patients with heterotopic interstitial pregnancy treated by LA and found that all patients were full-term delivery except one without obstetric data (15). Jeong et al. summarized the obstetric outcomes of the laparoscopic approach for 17 HP patients. The authors stated that 13 delivered 14 healthy babies, and only two failed to maintain their pregnancies (30). Li et al. also reported the pregnancy outcome of laparoscopic cornual resection for 8 HP patients, and found that LA may be a potential risk factor for abortion with a miscarriage rate (12.5%) (21). The evidence of inconsistent results found in these studies were limited due to its small sample size and without comparison with open surgery.

In this study, we found that compared with OA, the LA demonstrated less intraoperative blood loss and fewer days of hospital stay, which reflected the advantages of laparoscopic minimally invasive therapy. Although LA showed shorter hospital days, the hospital stays in our study were relatively longer than that in western countries, may be mainly explained by the fact that these women were kept hospitalized for fetal surveillance. Because most of women had the history of miscarriage or ectopic pregnancy or conceived *via* ART and thus experienced great emotional burdens. Taking into account of concerns for miscarriage after surgery, women are willing to require prolonged hospitalization. Besides, this may be related to Chinese traditional customs of tocolysis. A similar hospital stay of HP patients was reported in another study from China (31).

In addition, compared with OA, LA demonstrated less intraoperative bleeding. This finding is expected due to larger surgical incisions and more urgent situation in OA increasing the possibility of blood loss. Despite this, the total amount of intraoperative bleeding in both surgical methods was little, which could be reflected by 76 (88%) cases with bleeding less than 50 ml and the similar changes in hemoglobin levels before and after surgery. In our study, no statistically significant difference in operative time between two types of surgical methods was found, which is consistent with existing studies on adnexal masses or torsion and interstitial pregnancy treated by LA and OA (32–35). With the standardization of laparoscopic surgery and accumulation of experience, the operative time of LA has been significantly reduced in recent years. Besides, often experienced, the operating time was increased by larger surgical incisions for patients underwent OA. Thus, we found similar operative time between LA and OA groups.

In this study, the average total cost for LA was relatively higher than that of OA. It may be caused by the additional instruments and corresponding expenditures needed in LA. However, laparoscopy may actually have greater economic advantages because of the quicker recovery and earlier back to work from a long-term perspective.

For HP treatment, the safety of intrauterine fetus is another concern. Clayton et al. (7) claimed surgical management of the EP could increase the likelihood of intrauterine abortion. In our study, most women (81/86, 94%) had successful delivery for live birth, which was consistent with previous studies (31, 36, 37). Our HP live birth rate was higher than the live birth rate (66%–69%) reported by other studies (5, 8, 38). The ratio of first trimester miscarriage after the operation was only 6% (4/62) and 4% (1/24) in LA and OA, respectively. The high live birth rate in our study may be related to the timely diagnosis and intervention by experienced doctors. Moreover, no significant differences of pregnancy outcomes (first trimester miscarriage, birth weight, cesarean section rate and Apgar score) were found between the LA and the OA groups. Our results suggested the similar pregnancy outcome in patients with LA or OA for treating HP.

The strength of our work is the first study with a relatively large sample size to compare the two surgical approaches on perioperative and pregnancy outcomes in the treatment of HP for patients after embryo transfer by taking into account of its rarity. Compared with existed case reports and case-series studies, this study could provide a relatively higher level of evidence for clinician to choose laparoscopic surgery for those patients with HP. Besides, this study could provide some insight to focus on the efficacy and safety of different types of laparoscopy, such as a natural orifice transluminal endoscopic surgery (NOTES) reported in a case report (39), in the future.

The present study also had some limitations. First, it's a retrospective study. However, the data from medical records was collected objectively, which may to some extent reduce the retrospective bias. Frankly, it's extremely difficult to carry out a prospective study due to the rarity of this disease. In addition, the findings may not be generalizable due to the single-center design of this study, as well as sociocultural aspects and health care policies. Therefore, more studies from different centers are needed to validate our results.

## Conclusions

Laparoscopy maybe a safe and feasible procedure for treating HP patients. Compared with open approach, it showed a more favorable perioperative outcomes and similar pregnancy outcomes. If surgery is indicated and conditions permit, Laparoscopy could be a preferred method for the treatment of patients with HP.

## Data Availability

The original contributions presented in the study are included in the article/Supplementary Material, further inquiries can be directed to the corresponding author/s.
